# A New Strategy to Identify and Annotate Human RPE-Specific Gene Expression

**DOI:** 10.1371/journal.pone.0009341

**Published:** 2010-05-07

**Authors:** Judith C. Booij, Jacoline B. ten Brink, Sigrid M. A. Swagemakers, Annemieke J. M. H. Verkerk, Anke H. W. Essing, Peter J. van der Spek, Arthur A. B. Bergen

**Affiliations:** 1 Department of Clinical and Molecular Ophthalmogenetics, Netherlands Institute for Neuroscience, Royal Netherlands Academy of Arts and Sciences, Amsterdam, The Netherlands; 2 Department of Bioinformatics and Genetics, Erasmus Medical Center, Rotterdam, The Netherlands; 3 Cancer Genomics Centre, Erasmus Medical Center, Rotterdam, The Netherlands; 4 Clinical Genetics Academic Medical Centre Amsterdam, University of Amsterdam, The Netherlands; 5 Department of Ophthalmology, Academic Medical Centre Amsterdam, University of Amsterdam, The Netherlands; University of Washington, United States of America

## Abstract

**Background:**

To identify and functionally annotate cell type-specific gene expression in the human retinal pigment epithelium (RPE), a key tissue involved in age-related macular degeneration and retinitis pigmentosa.

**Methodology:**

RPE, photoreceptor and choroidal cells were isolated from selected freshly frozen healthy human donor eyes using laser microdissection. RNA isolation, amplification and hybridization to 44 k microarrays was carried out according to Agilent specifications. Bioinformatics was carried out using Rosetta Resolver, David and Ingenuity software.

**Principal Findings:**

Our previous 22 k analysis of the RPE transcriptome showed that the RPE has high levels of protein synthesis, strong energy demands, is exposed to high levels of oxidative stress and a variable degree of inflammation. We currently use a complementary new strategy aimed at the identification and functional annotation of RPE-specific expressed transcripts. This strategy takes advantage of the multilayered cellular structure of the retina and overcomes a number of limitations of previous studies. In triplicate, we compared the transcriptomes of RPE, photoreceptor and choroidal cells and we deduced RPE specific expression. We identified at least 114 entries with RPE-specific gene expression. Thirty-nine of these 114 genes also show high expression in the RPE, comparison with the literature showed that 85% of these 39 were previously identified to be expressed in the RPE. In the group of 114 RPE specific genes there was an overrepresentation of genes involved in (membrane) transport, vision and ophthalmic disease. More fundamentally, we found RPE-specific involvement in the RAR-activation, retinol metabolism and GABA receptor signaling pathways.

**Conclusions:**

In this study we provide a further specification and understanding of the RPE transcriptome by identifying and analyzing genes that are specifically expressed in the RPE.

## Introduction

The retinal pigment epithelium (RPE) is a monocellular retinal layer that plays a particularly important role in visual function. This is illustrated by its involvement in a large number of severe retinal disorders like age-related macular degeneration and retinitis pigmentosa.

**Figure 1:**
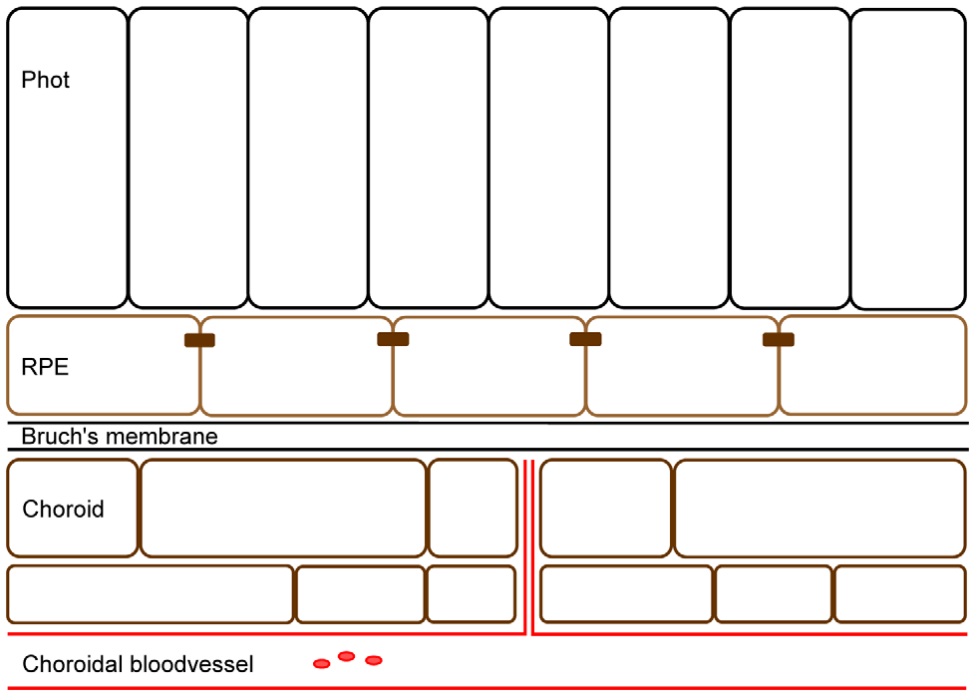
Overview of the (cell) layers surrounding the RPE. The dark brown rectangles connecting the RPE cells indicate tight junctions present between the cells. Phot: photoreceptors.

The RPE has multiple functions including supplying the photoreceptors with nutrients, recycling retinal from the photoreceptors and regulating the ion balance in the subretinal space. The RPE secretes a number of growth factors. Thereby it is involved in the maintenance of the structure and cellular differentiation of adjacent (cell) layers, the photoreceptors on the apical side, and Bruch's membrane and the choroid on the basolateral side (See Figure 1) [1].

Embryologically, both the RPE and the photoreceptors develop from the neuro-epithelium. A fold in the neuro-epithelium causes two layers of this structure to face each other [1]. One of these layers develops into the RPE, the other into the neural retina. The neural retina further differentiates and photoreceptors develop in close interaction with the microvilli of the RPE [1]. The development of the choroid from neural crest cells also depends on cues from the mature RPE [2]. The choroidal vasculature is created through angiogenesis from existing blood vessels from the paraocular mesenchyme [2]. Finally, Bruch's membrane (BM) is created from the basement membrane of the RPE and the basement membrane of the endothelium. In this way, the retina develops into a neatly arranged multi-layered structure (Figure 1).

We recently analyzed the RPE transcriptome [3]. We reported on the expressed genes and correlated molecular pathways in the RPE from cells that were specifically isolated from healthy human donor eyes [3]. Functional annotation of the RPE transcriptome showed that the RPE has high levels of protein synthesis, strong energy demands, is exposed to high levels of oxidative stress and has a variable degree of inflammation [3]. These data confirmed and expanded our knowledge on functional properties of the RPE, previously identified in a number of studies [4]–[7]. Nonetheless, due to the study design, our previous study was limited by unavoidable contamination from adjacent cell types [3], [5], [8].

In the current study we have overcome this limitation by using a new 44 k microarray strategy that includes both the RPE cell layer and the adjacent cell layers in the experiment. We compared the transcriptomes of the photoreceptor, RPE and choroidal cells using a single platform. We deduced that at least 114 genes are specifically expressed in the RPE and we describe their corresponding pathways.

## Results

We performed microarray analyses on RNA specifically isolated from RPE, photoreceptors and choroid cells from healthy human donor eyes. Recently, we defined the RPE transcriptome by measuring gene expression levels and identifying functional properties of the RPE [3]. The current study provides a further specification of the RPE transcriptome by identifying genes expressed at much higher levels in the RPE than in either adjacent cell layer, the photoreceptors and the choroid. In addition, we expand our dataset from a 22 k microarray platform to a 44 k microarray platform, resulting in a more extensive coverage of the human genome [9], and we used more advanced bioinformatics to analyze the data.

### RPE Gene Expression Compared to either Photoreceptors or Choroid

Of the 33,712 features present on the microarray (GSE20191 [10], see Text S1), 1,904 (5.6%) genes had at least 2.5-fold higher expression in the RPE than in the photoreceptors (RPE>phot) on average over three arrays (see Table 1 and Text S2). There was a significant overrepresentation of genes that encode signal proteins, glycoproteins, secreted proteins, membrane proteins, cell adhesion proteins, extracellular matrix proteins, proteins involved in the immune response, Ca^2+^-binding and actin binding (David [11]). Functional analysis of these genes revealed an overrepresentation of genes in the following pathways: cell adhesion molecules, melanogenesis and type I diabetes mellitus.

**Table 1:** Top 30 genes with highest expression in RPE compared to photoreceptors [27], [30].

Gene symbol	Genbank ID	FC RPE/chor&phot
*ITGB8*	BC042028	44
*C1orf168*	AK093468	41
*COL8A1*	AL359062	37
*SLC26A4*	NM_000441	37
*SLC26A7*	NM_052832	37
*DKFZp761G0122*	AL713743	37
*CLIC6*	NM_053277	34
*VASN*	NM_138440	33
*SMOC2*	NM_022138	31
*SLC16A12*	AK124901	29
*C6orf105*	NM_032744	28
*SLC6A13*	BC020867	28
*PRDM16*	NM_022114	28
*LGI1*	NM_005097	27
*PLD5*	NM_152666	26
*A_32_P114831*	A_32_P114831	26
*KCNS3*	NM_002252	25
*BEST1*	NM_004183	23
*IL8*	NM_000584	23
*TMEM27*	NM_020665	23
*NTN4*	NM_021229	23
*RWDD3*	AK126344	23
*LRP8*	NM_004631	23
*SLCO1C1*	NM_017435	23
*COL8A2*	NM_005202	22
*MYRIP*	NM_015460	22
*GPNMB*	NM_002510	20
*PLA2G7*	NM_005084	20
*KCNJ13*	NM_002242	19
*FAM40B*	AB032996	19

FC: fold change, average expression level in RPE compared to choroid and photoreceptors in six arrays.

Furthermore, 1,126 (3.3%) of 33,712 genes on the array had at least 2.5-fold higher expression in the RPE than in the choroid (RPE>chor) on average over three arrays (see Table 2 and Text S3). In this group of genes there was a significant overrepresentation of genes coding for proteins involved in vision, retinitis pigmentosa and (membrane) transport, as well as genes with a symport function and genes in the olfactory transduction pathway.

**Table 2:** Top 30 genes with highest expression in RPE compared to choroid [27], [30].

Gene symbol	Genbank ID	FC RPE/chor&phot
*RBP3*	NM_002900	37
*RPE65*	NM_000329	24
*MPP4*	NM_033066	24
*ELOVL4*	NM_022726	23
*GUCA1C*	NM_005459	23
*PDE6G*	NM_002602	22
*NEUROD1*	NM_002500	20
*BEST1*	NM_004183	20
*SLC6A13*	BC020867	19
*ITGB8*	BC042028	19
*RP1*	NM_006269	19
*GUCA1B*	BX537393	19
*PDC*	NM_002597	19
*CNGB3*	NM_019098	18
*PROM1*	NM_006017	18
*PRPH2*	NM_000322	18
*HCN1*	AK094523	18
*DKFZp761G0122*	AL713743	17
*HOOK1*	AK027250	17
*GNAT2*	NM_005272	16
*RLBP1*	NM_000326	16
*C6orf105*	NM_032744	16
*CNGA1*	NM_000087	16
*ABCA4*	NM_000350	16
*TMEM27*	NM_020665	16
*RWDD3*	AK126344	16
*OPCML*	BX537377	15
*NRL*	NM_006177	15
*C1orf168*	AK093468	15
*TMEM16B*	NM_020373	15

Note that cellular contamination of the RPE cells may be present, identified by the *ABCA4* transcript, which is truly a photoreceptor-specific transcript [31]. FC: fold change, average expression level in RPE compared to choroid and photoreceptors in six arrays.

### A Novel Strategy to Detect RPE-Specific Gene Expression

Until now, all RPE transcriptome studies, including our own [3], were aimed at excluding the cell layers adjacent to the RPE from the experiment in order to prevent cellular contamination and to achieve the highest RPE tissue specificity possible [3], [4]. However even isolation of RPE cells by meticulous laser dissection microscopy resulted in unavoidable contamination with adjacent cell types [5], [8] (this study).

In the current study however, we did not discard the adjacent cell layers as possible contaminants, but we included them as valuable resources for comparison of gene expression. Our primary objective was to further specify the expression level of genes in the RPE relative to their expression in the photoreceptors and the choroid. In this way, we deduced RPE-specific gene expression.

### RPE-Specific Gene Expression

As a first analysis we identified all genes with *average* expression levels *at least* 2.5-fold higher in the RPE than in both photoreceptors and choroid. This resulted in a list of 458 entries with RPE-specific expression (see Text S4). Functional annotation showed an overrepresentation of genes involved in inositol metabolism, retinol metabolism, genetic disorders and ophthalmic diseases (data not shown).

In order to illustrate the usefulness of our approach to identify RPE specific transcripts, we next employed even stricter criteria (i.e. in *all* six arrays *at least* 2.5-fold higher expression levels in the RPE than in both photoreceptors and choroid (RPE>phot&chor FC>2.5)). This yielded 114 genes (see Table 3). We deduced from our recently published data set [3] that 39 out of these 114 genes are very highly expressed in the RPE (see Table 4).

**Table 3:** 114 genes with at least 2.5 fold higher RPE expression in the RPE than in both the photoreceptors and the choroid in all six microarrays, defined as RPE-specific expression.

Gene symbol	Genbank ID	FC	gene symbol	Genbank ID	FC	gene symbol	Genbank ID	FC
*ITGB8*	BC042028	31	*SLC16A14*	NM_152527	10	*ACOT11*	NM_147161	7
*C1orf168*	AK093468	28	*WFDC1*	NM_021197	10	*CDH3*	NM_001793	6
*DKFZp761G0122*	AL713743	27	*SLC6A13*	NM_016615	10	*FRZB*	NM_001463	6
*SLC6A13*	BC020867	24	*CACNB2*	BG428517	10	*LOC439949*	AY007155	6
*C6orf105*	NM_032744	22	*SLC2A12*	NM_145176	10	*SERPINF1*	NM_002615	6
*CLIC6*	NM_053277	22	*SLC6A12*	NM_003044	10	*GPAM*	NM_020918	6
*BEST1*	NM_004183	21	*KIAA0953*	AF131834	10	*SPOCK1*	NM_004598	6
*RPE65*	NM_000329	20	*ADORA2B*	NM_000676	10	*FLJ30594*	NM_153011	6
*PLD5*	NM_152666	19	*CA14*	NM_012113	10	*MUPCDH*	NM_031264	6
*TMEM27*	NM_020665	19	*PNPLA3*	NM_025225	10	*C1orf168*	AK125198	6
*RWDD3*	AK126344	19	*RGR*	NM_002921	10	*CLDN19*	NM_148960	6
*LRP8*	NM_004631	19	*STRA6*	NM_022369	9	*LMO1*	NM_002315	6
*LGI1*	NM_005097	18	*C7orf46*	NM_199136	9	*GLDC*	NM_000170	6
*SLC16A12*	AK124901	17	*KIRREL2*	NM_199180	9	*A_24_P186746*	A_24_P186746	5
*FAM40B*	AB032996	17	*RDH5*	NM_002905	9	*RDH11*	NM_016026	5
*PRDM16*	NM_022114	16	*BMP4*	NM_001202	9	*SFRP5*	NM_003015	5
*MYRIP*	NM_015460	16	*TMEM56*	NM_152487	9	*SGK1*	NM_005627	5
*BMP7*	NM_001719	15	*THC1892753*	THC1892753	9	*KRT18*	NM_000224	5
*ERMN*	AB033015	14	*CNKSR3*	NM_173515	9	*OPHN1*	NM_002547	5
*SLC13A3*	NM_022829	14	*CCNO*	NM_021147	8	*TDRD9*	NM_153046	5
*SLCO1C1*	NM_017435	14	*RDH10*	NM_172037	8	*EZR*	NM_003379	5
*LRAT*	NM_004744	13	*PBX4*	NM_025245	8	*FAM40B*	BC019064	5
*OPCML*	BX537377	13	*SKIP*	NM_130766	8	*C7orf46*	BC042034	5
*RLBP1*	NM_000326	13	*SLC7A10*	NM_019849	8	*CTSD*	AK022293	5
*TRPM3*	NM_206948	13	*CXCL14*	NM_004887	8	*DHCR7*	NM_001360	4
*KCTD4*	NM_198404	13	*A_24_P234871*	A_24_P234871	8	*ITGAV*	NM_002210	4
*THC1934449*	THC1934449	13	*COL20A1*	NM_020882	8	*GALNT11*	NM_022087	4
*LRP8*	NM_017522	12	*LHX2*	NM_004789	8	*THC1967593*	THC1967593	4
*SLC39A12*	NM_152725	12	*C1QTNF5*	NM_015645	7	*LOC650392*	BC036550	4
*DUSP6*	NM_001946	12	*SLC22A8*	NM_004254	7	*HPD*	NM_002150	4
*ERMN*	BC026345	11	*ROBO2*	AK074780	7	*BCAT1*	NM_005504	4
*SLCO1A2*	NM_005075	11	*THC1970019*	THC1970019	7	*A_23_P122650*	A_23_P122650	4
*RBP1*	NM_002899	11	*ADAD2*	NM_139174	7	*A_32_P226525*	A_32_P226525	4
*SLC16A3*	NM_004207	11	*OR51E2*	NM_030774	7	*PCP4*	NM_006198	4
*CNDP1*	NM_032649	11	*SLC6A20*	NM_020208	7	*A_32_P112546*	A_32_P112546	4
*SLCO1A2*	NM_134431	11	*SLC16A8*	NM_013356	7	*KIAA1576*	NM_020927	4
*THC1839330*	THC1839330	11	*THC2004763*	THC2004763	7	*A_23_P73096*	A_23_P73096	4
*SULF1*	NM_015170	11	*A_24_P109661*	A_24_P109661	7	*BASP1*	NM_006317	3

FC: fold change, average expression level in RPE compared to choroid and photoreceptors in six arrays.

**Table 4:** 39 RPE-specific genes with high expression levels as determined in our previous study [3].

gene symbol	Genbank ID	FC RPE/chor&phot	RPE expression in literature	ref	review Schulz
*C6orf105*	NM_032744	22	microarray	[32]	
*BEST1*	NM_004183	21	immunohistochemical staining	[33]	
*TMEM27*	NM_020665	19	this study, Figure 11		
*LRP8*	NM_004631	19	microarray	[32]	1
*LGI1*	NM_005097	18	review, exclusively in RPE studies	[4]	1
*FAM40B*	AB032996	17	cDNA clones	[34]	
*ERMN*	AB033015	14	this study, Figure 11		1
*LRAT*	NM_004744	13	western blot, northern blot	[35]	
*RLBP1*	NM_000326	13	fluorescence immunocytochemistry	[36]	1
*DUSP6*	NM_001946	12	est database	[37]	1
*RBP1*	NM_002899	11	review, genes expressed in retina/RPE	[4]	1
*SLC16A3*	NM_004207	11	immunofluorescence	[38]	1
*WFDC1*	NM_021197	10	immunocytochemistry, microarray	[8], [39]	1
*KIAA0953*	AF131834	10	review, genes expressed in retina/RPE	[4]	
*CA14*	NM_012113	10	immunocytochemistry	[40]	1
*RGR*	NM_002921	10	microarray	[32]	1
*STRA6*	NM_022369	9	immunohistochemistry	[41]	
*RDH5*	NM_002905	9	northern blot	[20]	1
*BMP4*	NM_001202	9	RNAse protection assay	[42]	1
*CXCL14*	NM_004887	8	microarray, RT-PCR	[39]	1
*LHX2*	NM_004789	8	in situ hybridization	[43]	1
*C1QTNF5*	NM_015645	7	cDNA library, RT-PCR	[44], [45]	1
*SLC6A20*	NM_020208	7	this study, Figure 11		
*SLC16A8*	NM_013356	7	PCR	[46]	1
*CDH3*	NM_001793	6	western blot	[47]	1
*FRZB*	NM_001463	6	review, genes expressed in retina/RPE	[4]	1
*SERPINF1*	NM_002615	6	amino acid sequencing	[48]	1
*SPOCK1*	NM_004598	6	this study, Figure 11		
*LMO1*	NM_002315	6	this study, Figure 11		
*RDH11*	NM_016026	5	bovine and monkey, immunohistochemistry and in situ hybridization	[21]	1
*SFRP5*	NM_003015	5	northern blot	[49]	1
*SGK1*	NM_005627	5	this study, Figure 11		1
*KRT18*	NM_000224	5	CNV RPE RT-PCR	[50]	1
*EZR*	NM_003379	5	rat immunofluorescence and immunoelectron microscopy	[51]	1
*DHCR7*	NM_001360	4	microarray	[32]	1
*ITGAV*	NM_002210	4	microarray	[32]	1
*GALNT11*	NM_022087	4	review, genes expressed in retina/RPE	[4]	1
*PCP4*	NM_006198	4	review, genes expressed in retina/RPE	[4]	1
*BASP1*	NM_006317	3	microarray	[32]	1

Column four and five show 33 of the 39 genes (85%) were previously described (RNA or protein level) in individual studies in the literature using several different techniques to have RPE expression. Column six shows that 29 of the 39 genes (74%) were also present in a study by Schulz et al. [4] reporting on 13,037 genes expressed in the retina/RPE (their supplementary table 2) ref: literature reference, FC: fold change, average expression level in RPE compared to choroid and photoreceptors in six arrays, 1 in column six: present.

We next hypothesized that these 39 genes, given their very high expression, could easily have been detected by other means in previous studies. We compared our 39 highly expressed RPE-specific genes from the current study to a database containing 13,037 genes described to be expressed specifically in either the retina or the RPE, described by Schulz et al [4] and were able to identify 29 of our genes in their database (75%) (see Table 4). A more thorough manual search of individual studies on the expression of genes in the RPE showed that 85% of the 39 genes were previously identified to be expressed in the RPE (Table 4).

**Figure 2:**
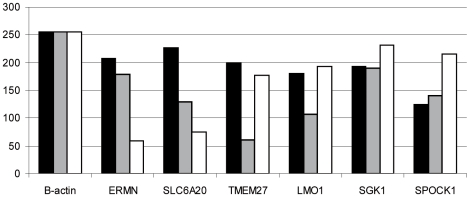
Confirmation of microarray results by s-QPCR. Beta actin, a household gene, was used to normalize gene expression in between all cells of the retina. The black bars indicate RPE expression levels, the grey bars indicate choroid expression and the empty bars indicate photoreceptor expression. For all genes RPE expression was shown in the microarray. See also text and Table 4.

For the remaining 15% (6 genes) no data was available in the literature. Using semi quantitative QPCR (s-QPCR), we confirmed RPE expression of these genes (Table 4 and Figure 2). Both ERMN (AB033015) and SLC6A20 (NM_020208) appear to be more highly expressed in RPE compared to both choroid or photoreceptors, thereby fully confirming the microarray data. TMEM27 (NM_020665) and LMO1 (NM_002315) appear also to more highly expressed in the RPE than in the choroid, but show approximate equal expression in the photoreceptors. SGK1 (NM_005627) is ubiquitously expressed in all three cells examined. The expression of SPOCK1 (NM_004598) in the RPE is rather low compared to the photoreceptors and does apparantly not confirm the microarray data.

### Functional Annotation of RPE-Specific Gene Expression

**Figure 3:**
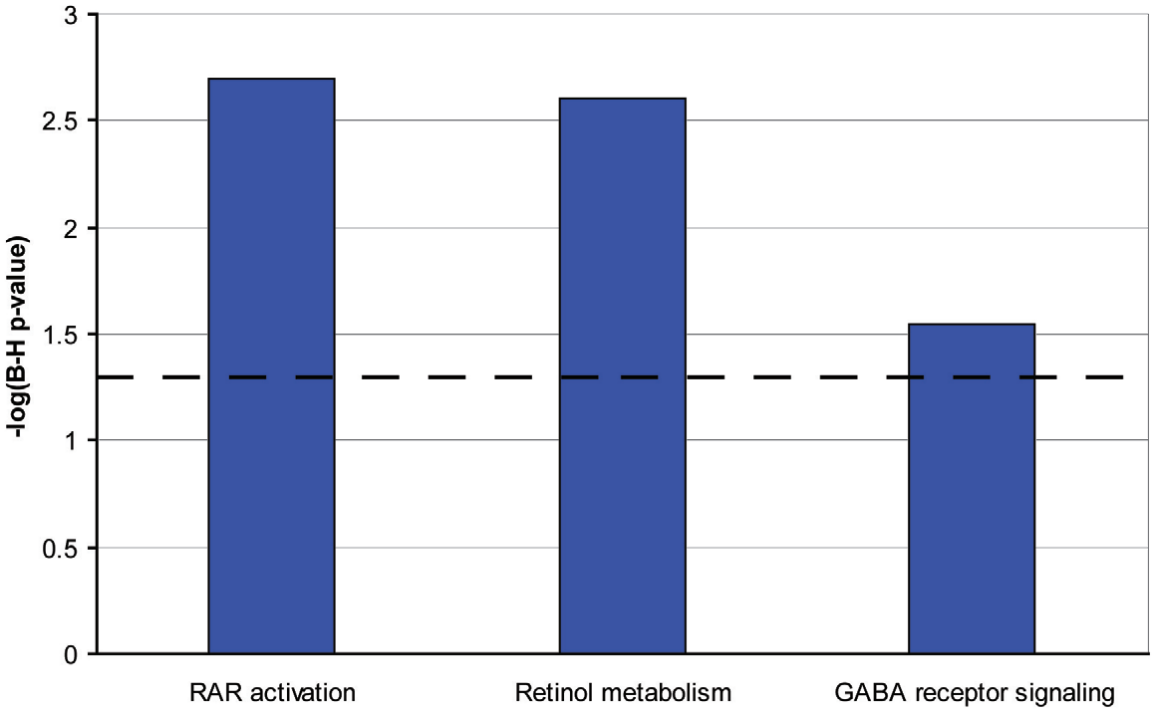
Three canonical pathways identified by Ingenuity software [**25**] in the group of RPE-specific genes. The three bars represent the canonical pathway identified, the x-axis identifies the pathways. The y-axis shows the −log of the Benjamini-Hochberg (B–H) p-value. The dotted line represents the threshold above which there are statistically significantly more genes in a pathway than expected by chance.

**Figure 4:**
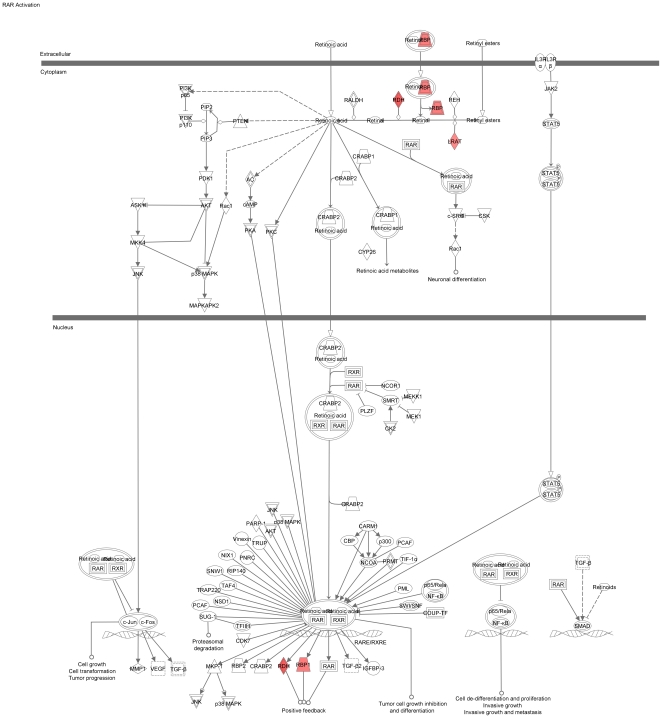
The RAR-activation pathway identified by the Ingenuity software. This is one of the canonical pathways that contains statistically significantly more genes than expected by chance in the group of 114 genes with RPE-specific expression. This figure shows the proteins corresponding to the overrepresented genes. Colored fields indicate their presence among the 114 genes with RPE-specific expression, uncolored genes are added by the software to form pathways. Solid lines between molecules indicate direct physical relationships between molecules (such as regulating and interacting protein domains); dotted lines indicate indirect functional relationships (such as co-regulation of expression of both genes in cell lines).

**Figure 5:**
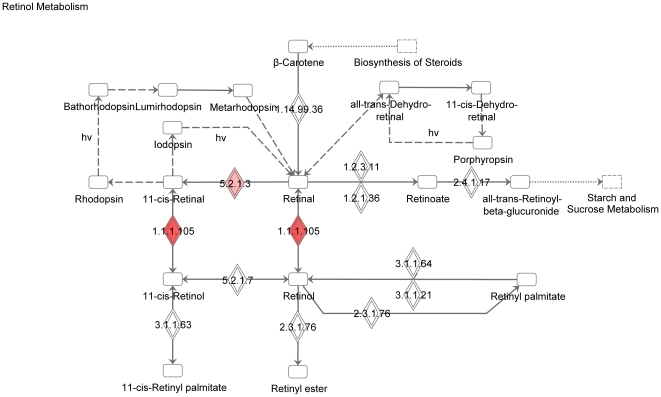
The retinol metabolism pathway identified by the Ingenuity software. This is one of the canonical pathways that contains statistically significantly more genes than expected by chance in the group of 114 genes with RPE-specific expression. This figure shows the proteins corresponding to the overrepresented genes. Colored symbols indicate their presence among the 114 genes with RPE-specific expression, uncolored genes are added by the software to form pathways. Solid lines between molecules indicate direct physical relationships between molecules (such as regulating and interacting protein domains); dotted lines indicate indirect functional relationships (such as co-regulation of expression of both genes in cell lines).

**Figure 6:**
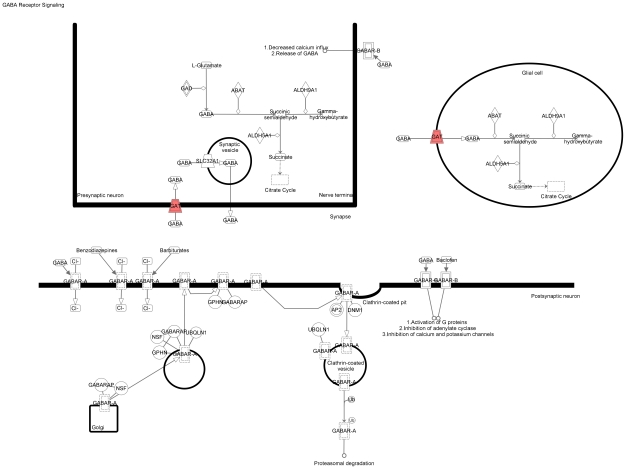
The GABA receptor signaling pathway identified by the Ingenuity software. This is one of the canonical pathways that contains statistically significantly more genes than expected by chance in the group of 114 genes with RPE-specific expression. This figure shows the proteins corresponding to the overrepresented genes. Colored symbols indicate their presence among the 114 genes with RPE-specific expression, uncolored genes are added by the software to form pathways. Solid lines between molecules indicate direct physical relationships between molecules (such as regulating and interacting protein domains); dotted lines indicate indirect functional relationships (such as co-regulation of expression of both genes in cell lines).

**Figure 7:**
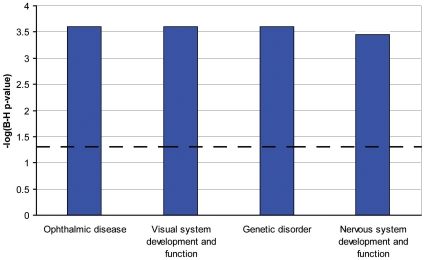
Four most significant biological functions identified by Ingenuity software [**25**] in the group of RPE-specific genes. The four bars represent the canonical pathway identified, the x-axis identifies the pathways. The y-axis shows the −log of the Benjamini-Hochberg (B–H) p-value. The dotted line represents the threshold above which there are statistically significantly more genes in a pathway than expected by chance.

**Figure 8:**
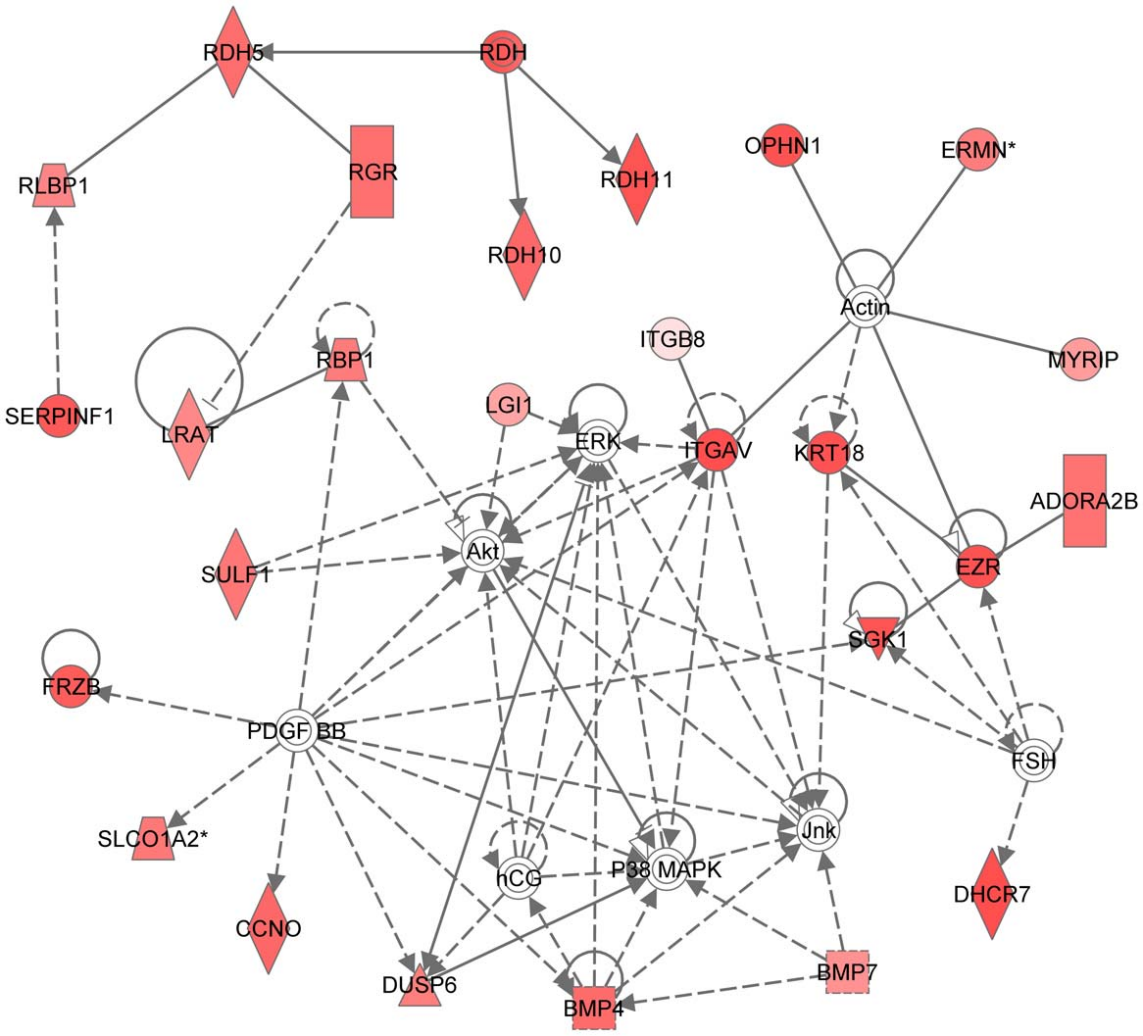
Most significant molecular network generated by the Ingenuity software. Network is generated from our dataset with RPE-specific expression (114 entries; see text). Note that the colored symbols represent gene entries that occur in our data set, while the transparent entries are molecules from the knowledge database, inserted to connect all relevant molecules in a single network. Solid lines between molecules indicate direct physical relationships between molecules (such as regulating and interacting protein domains); dotted lines indicate indirect functional relationships (such as co-regulation of expression of both genes in cell lines). Abbreviation of gene names are according to standard abbreviations used in Genbank [29]. The main functionalities given by Ingenuity for this molecular network are Nervous system development and function, Visual system development and function, organismal development. This network overlaps with the network in Figure 2). Highlights in this network include: (a) the regulating role of the MAPK/ERK pathway, a very complex signal transduction pathway that couples intracellular responses to the binding of growth factors to cell surface receptors; (b) platelet-derived growth factor (PDGF BB), known to induce RPE cell proliferation and migration and the development of proliferative vitreoretinopathy (PVR), acts indirectly on multiple molecules in this network, and c) the RPE retinol metabolism is present in the periphery of this molecular network.

**Figure 9:**
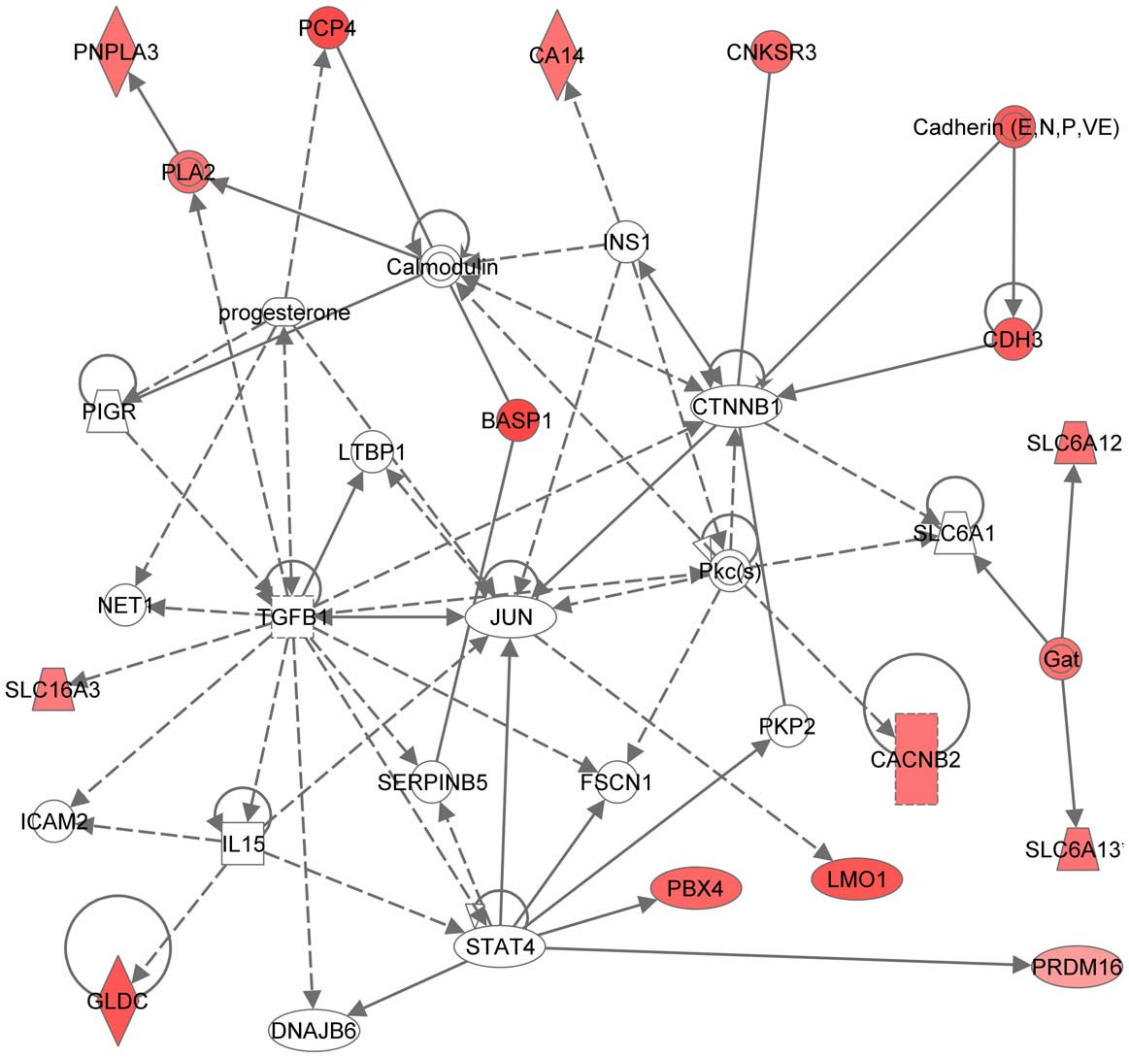
Second most significant molecular network generated by the Ingenuity software. Network is generated from our microarray dataset with RPE-specific expression (114 entries; see text). For explanation of symbols on the diagrams see legend Figure 7. The main functionalities given by Ingenuity for this molecular network are cellular development, hematological system development and function and connective tissue development and function. Highlights in this network include: (a) The dual presence of GABA receptors *SLC6A12* [NM_003044] and *SLC6A13* [NM_016615]; the presence and interactions of (b) the insulin-1 (INS1) protein and (c) the hormone progesterone.

**Figure 10:**
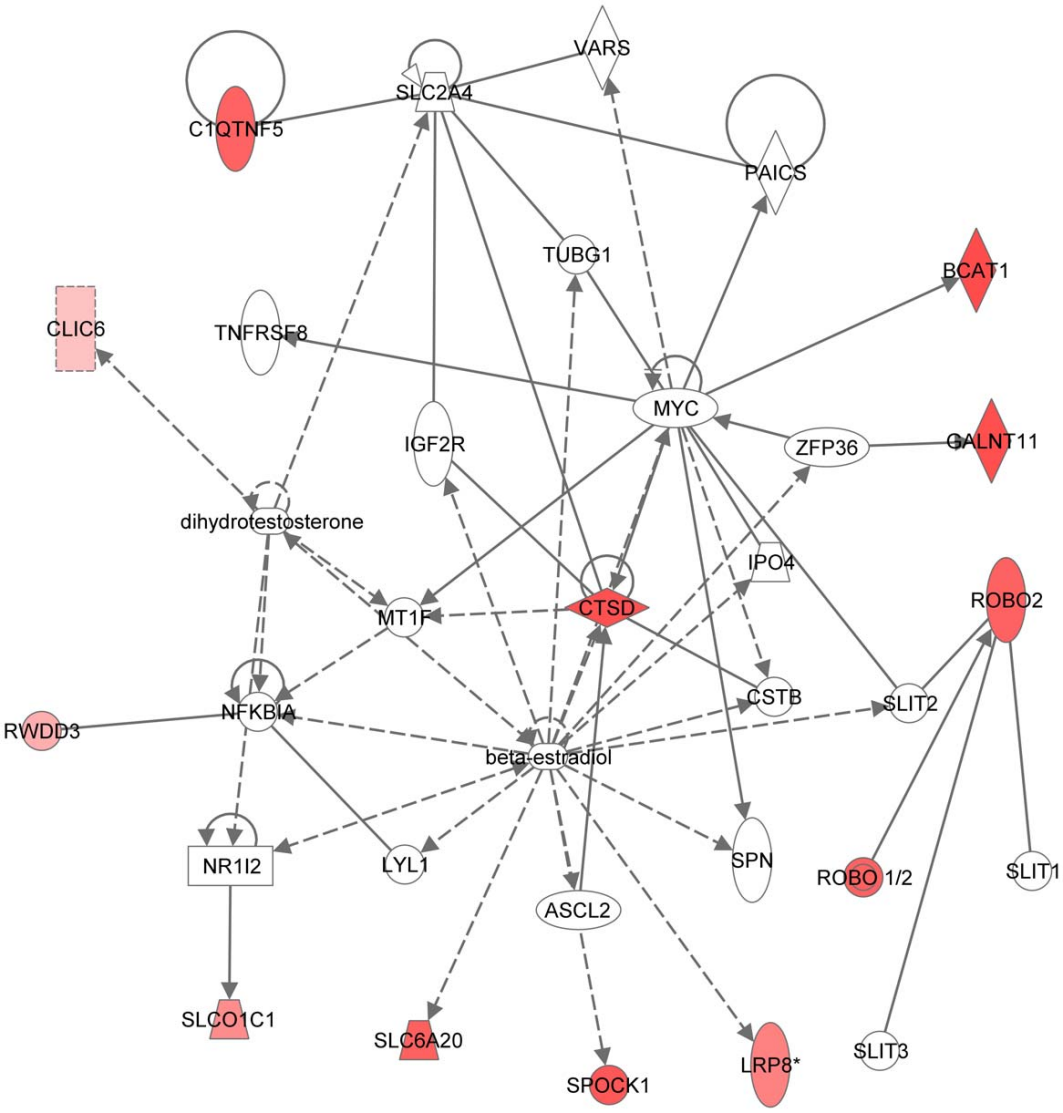
Third significant molecular network generated by Ingenuity. Network is generated from our microarray dataset with RPE-specific expression (114 entries; see text). For explanation of symbols on the diagram see legend Figure 7. The main functionalities given by Ingenuity for this molecular network are cellular movement, nervous system development and function and gene expression. Please note the central signaling role of beta-estradiol; known to affect retinal function and disease.

**Figure 11:**
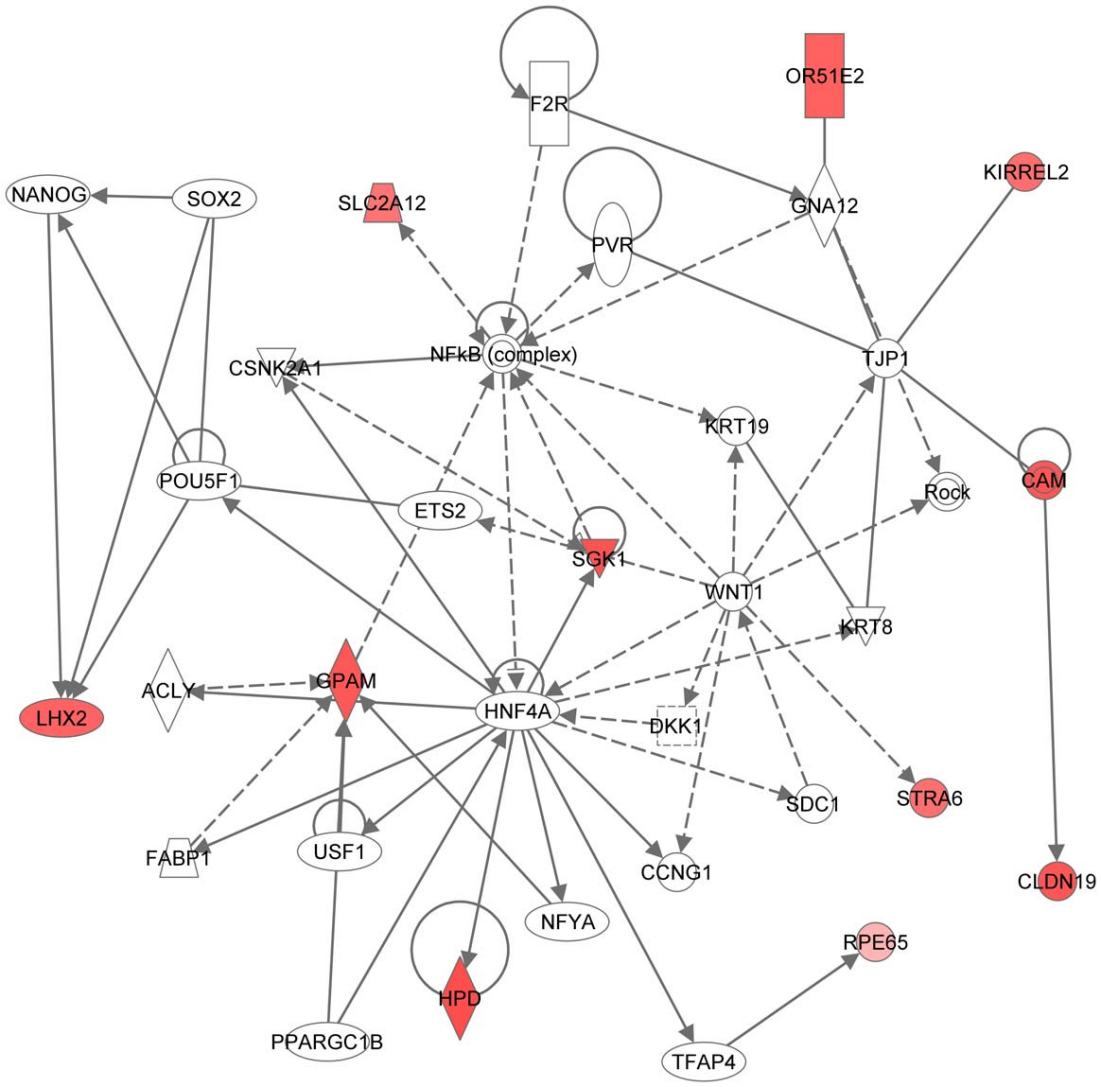
Fourth significant molecular network generated by Ingenuity. Network is generated from our microarray dataset with RPE-specific expression (114 entries; see text). For explanation of symbols on the diagrams see legend Figure 7. This network overlaps with the network in Figure 7. The main functionalities given by Ingenuity for this molecular network are lipid metabolism, molecular transport and nucleic acid metabolism. The highlights of this network include: The central roles for (a) the *HNF4A* transcription factor and (b) the NFkappa Beta and (c) Wnt signaling pathways.

We used the list of 114 RPE-specifically expressed genes for further functional annotation of the major pathways specific for the RPE. The online database DAVID did not identify any Kegg pathways in this group of genes. However, among the 95 genes recognized by the Ingenuity software, there was a significant overrepresentation of genes in the following functional categories: symport, membrane transport, vision, glycoprotein, transport (p<0.0001). Moreover, using the Ingenuity database we identified three canonical pathways (see Figure 3): RAR-activation (Figure 4), retinol metabolism (Figure 5) and GABA receptor signaling (Figure 6). We found four biological functions with a significant overrepresentation of genes: ophthalmic disease, visual system development and function, genetic disorder and nervous system development and function (see Figure 7). Finally, we identified four networks correlated with our RPE-specific gene list (see Figures 8, 9, 10, and 11).

Similar functional annotations were found for the group of 39 highly expressed RPE-specific genes (data not shown). Upon closer inspection of this group we also identified eight (almost one in five!) known retinal disease genes (*BEST1* [NM_004183], *C1QTNF5* [NM_015645], *CDH3* [NM_001793], *LRAT* [NM_004744], *RDH5* [NM_002905], *RDH11* [NM_016026], *RGR* [NM_002921], *RLBP1* [NM_000326] and *STRA6* [NM_022369]). Fifteen entries represented membrane bound or transmembrane genes.

## Discussion

### Study Design

In this study we provide a further specification of the RPE transcriptome, by analyzing the genes expressed in the RPE in reference to their expression in photoreceptors and choroid. We performed functional analyses on our microarray data using the online database DAVID and Ingenuity software in order to categorize the data and identify important functional properties in 114 genes specifically expressed in the RPE. Moreover, we identified 39 genes with RPE-specific expression and high expression levels in the RPE and in 85% of genes we were able to confirm RPE expression using the literature.

For the remaing 15%, sQPCRs were carried out which in most cases confirmed RPE enriched expression. Our current study design was focused on comparative gene expression, genes with high expression levels in two or multiple tissues were not included.

As discussed extensively elsewhere [5], [8], some degree of cellular contamination by applying LDM procedures to the retina is unavoidable. An important issue in this study was if we could overcome the cellular contamination problem by comparing the expression profiles of three adjacent cellular monolayers, in other words, did we succeed in identifying RPE specific transcripts? A number of considerations are important: 1) Obviously, if we would choose our comparative criteria even more strict (for example expression RPE >20 x than in photoreceptor or choroid, versus current criteria RPE >2.5 x than in photoreceptor or choroid), we would obtain an even more specific RPE expressed data set. 2) In our current dataset of 114 RPE genes, we did not find any obvious contamination of highly expressed (known) photoreceptor transcripts, like opsins. 3) The majority of the 114 genes interact with each other via a limited number of molecular pathways and networks, with functional annotations which more or less can can be attributed to the RPE. This further confirms the RPE specific origin of these transcripts. For the majority of completely unknown genes, our sQPCR data confirmed the microarray data. Only one entry (SPOCK1) showed apparent higher photoreceptor expression than the RPE equivalent, and did not confirm the microarray data. The reason for this discrepancy remains to be elucidated. All data taken together, RPE specificity for the majority of 114 transcripts identified is highly likely; better than previously, although some minor degree of contamination can still not be excluded.

### Functional Properties of the RPE

#### RPE versus photoreceptors only

Compared to the photoreceptors, the RPE cells express genes from three different functional categories at significantly higher levels. The first is cell adhesion molecules. The RPE represents the outer blood-retina barrier (BRB) and this group of molecules most likely illustrated the importance for the RPE to adhere firmly to BM in order to maintain the integrity of the barrier. The second pathway is the melanogenesis pathway uniquely present in the RPE. Indeed, in the heavily pigmented RPE, the pigment granules protect against oxidative stress [12]. The third RPE pathway is the type I diabetes mellitus pathway. Twelve out of 13 genes are members of the major histocompatibility complex (MHC). This pathway was also present among the genes with highly variable expression levels in the RPE in our previous study [3]. The MHC genes are responsible for antigen presentation and are implicated in the RPE specific immune response in both health and disease [13].

In addition to the Kegg pathways described above, there were genes overrepresented in a number of functional categories, related to major functions of the RPE. There were secreted proteins, proteins involved in signaling and Ca^2+^-binding proteins. It is well known that many RPE-specific Ca^2+^ channels are involved in intracellular signaling, cellular signal transduction and the regulation of secretion of various factors [14].

Additional functional categories included membrane proteins, cell adhesion proteins and extracellular matrix (ECM) proteins, that correlate with the structural role of the RPE and its interaction with BM [1], [15].

Finally, there was an overrepresentation of glycoproteins. Glycoproteins are crucial for the phagocytosis of photoreceptor outer segments [16].

#### RPE versus choroid only

In the group of genes with expression levels higher in the RPE than in the choroid, there was an overrepresentation of genes in the olfactory transduction pathway. This pathway contains mainly guanylate cyclases and calcium/calmodulin-dependent protein kinases, naturally present in such highly active cells as the RPE. In addition, there was a high abundance of genes involved in vision and transport. Obviously, the RPE plays a dominant role in the transport of many signaling molecules and in the transport of waste material from the photoreceptor cells.

#### Genes with RPE-specific expression

We functionally annotated the 114 genes with RPE-specific expression using both David and Ingenuity software. As expected, both programs yielded vision, visual and nervous system development and function, ophthalmic disease and genetic disorder as significantly overrepresented groups of genes. Indeed, most of the genes in which mutations lead to retinal disorders are genes with high and specific expression in either RPE or photoreceptors [3].

Using David we also found an overrepresentation of genes involved in sym- or transport. This most likely represents one of the major functions of the RPE, which is the transport of biomolecules from the choroid toward the photoreceptors and vice versa. The photoreceptors heavily depend on nutrients, oxygen, hormones, etc. from the bloodstream. Meanwhile, waste products like oxidized cholesterol, visual cycle intermediates and excess water leave the retina through the RPE [1].

The Ingenuity database also revealed three canonical pathways, RAR-activation, retinol metabolism and GABA receptor signaling. Binding of retinoic acid to the retinoic acid receptor (RAR), leads to tissue-specific activation or suppression of downstream transcription [17], [18]. In mature RPE, this process is invaluable for maintenance of differentiation and homeostasis [17]. The significant presence of this pathway may thus be explained by the need of the RPE to counteract local insults (oxidative stress, lipid digestion, lipofuscin accumulation) and maintain homeostasis.

The retinol metabolism pathway contained the *RDH5* [NM_002905], *RDH10* [NM_172037] and *RDH11* [NM_016026] genes, all three genes are involved in the RPE part of the visual cycle [19]. It is well known that the RPE converts all-*trans* retinoids to 11-*cis* isomers. More specifically, *RDH5* [NM_002905] and *RDH11* [NM_016026] convert 11-*cis* retinol to 11-*cis* retinal, while *RDH10* [NM_172037] converts 11-*cis* retina to all-*trans* retinal [19]–[22].

Finally, GABA is an important inhibitory neurotransmitter from the GABA receptor signaling pathway (Grsp) present in both brain and retina [23]. This pathway is involved in the retinal reuptake of GABA from the subretinal space. Interestingly, at least one protein from this pathway (SLC6A12 [NM_003044]) was previously observed in the rat and bullfrog RPE [23].

### Comparison of RPE-Expressed Genes to the Literature

Previous studies, combined into one review, claimed to reveal 246 genes to be expressed exclusively in the RPE [4]. Strikingly, in the current study, we found that 23 out of these 246 genes (9%) had higher expression levels in the photoreceptors than in the RPE. In addition, 72 of the 246 genes (29%) had higher expression levels in the choroid than the RPE. This indicates that at least a certain level of contamination is present in the RPE signal of previously performed studies. Consequently, care should be taken when interpreting these results.

### Conclusions

Our study provides a detailed description of RPE-specific gene expression, as compared to both adjacent cell layers, photoreceptors and the choroid. In addition we provide a detailed functional analysis of the functional properties of the RPE-specific genes. We show the involvement of the RPE in RAR-activation, retinol metabolism and GABA receptor signaling. Moreover, for 85% of the genes we call RPE-specific with high expression levels, we could more or less verify our results using the literature. Finally, we added a substantial number of new genes significantly expressed in the RPE.

## Methods

### Human Donor Eyes

This study was performed in agreement with the declaration of Helsinki on the use of human material for research. Material used in this study was provided to us by the Corneabank Amsterdam. In accordance with Dutch law, the Corneabank ensured none of the donors objected to the use of their eyes for scientific purposes. Approval of the medical ethics committee was not required as data were analyzed anonymously. A detailed description of our methods can be found elsewhere [3]. In brief, we selected five eyes from five human postmortem donors. Globes were enucleated between 16 and 22 hours post mortem and frozen several hours later according to a standard protocol. Donors were aged 63 to 78 years at time of death. We chose older donors in order to minimize the likelihood of the presence of yet undiagnosed monogenic eye diseases. The donors died of cardiovascular or cerebrovascular causes or of chronic obstructive pulmonary disease. Donors did not have a known ophthalmic disorder. Visual examination and histological examination, including periodic acid Schiff (PAS) staining, indicated no retinal pathology. Three eyes were selected for the analysis of RPE vs. choroid, due to limited tissue availability only one of these eyes was also used for the analysis of RPE vs. photoreceptors. For the second and third comparison of RPE vs. photoreceptors, two additional eyes were selected.

### Cell Sampling

**Figure 12:**
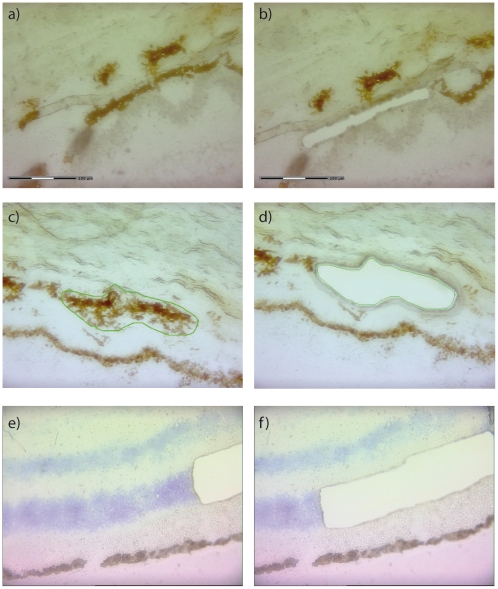
Frozen sections of the different cell types used in the study before and after laser dissection microscopy. Sections were used to isolate a) and b) RPE cells, c) and d) choroid, and e) and f) photoreceptor cells. Note the relatively poor morphology due to the use of frozen sections. The sections used for the isolation of photoreceptor cells were stained with cresyl violet (see methods section), the other sections were unstained. The scale can be found in figures a and b.

Globes were snap-frozen and stored at −80°C until use. A macular fragment of 16 mm^2^ with the fovea in its centre was cut from each of the retinas, as described previously [8]. For each eye, multiple cryosections were stained with periodic-acid Schiff and microscopically examined for abnormalities. Twenty µm sections from the macular areas were used for the isolation of choroid, RPE cells and photoreceptor cells. A Cresyl Violet staining (LCM Staining Kit, Ambion) was applied to the sections intended for the isolation of photoreceptor cells, according to the manufacturer's protocol. No staining was applied to sections to be used for the isolation of RPE cells or choroid. All sections were dehydrated with ethanol and air-dried before microdissection with a Laser Microdissection System (PALM, Bernried, Germany) (Figure 12). Cells were stored at −80° Celsius.

### RNA Isolation and Amplification

Total RNA was isolated and the mRNA component was amplified [8]. Amplified RNA (aRNA) was quantified with a nanodrop (Isogen Life Science B.V., The Netherlands) and the quality was checked on a BioAnalyzer (Agilent Technologies, Amstelveen, The Netherlands). Subsequently, aRNA samples were labeled with either a Cy3 or a Cy5 fluorescent probe.

### Microarray Design and Handling

For all hybridizations a 44 k microarray was used (Agilent Technologies, Amstelveen, The Netherlands). For three of the donors, photoreceptor RNA was hybridized against RPE RNA. In addition, for three donors, choroidal RNA was hybridized against RPE RNA. Hybridization, washing and scanning were performed as described previously [8].

### Data Analysis

Scanned images were processed and analyzed with Feature Extraction software (v 8.5 Agilent) and Rosetta Resolver software (Rosetta Inpharmatics). All data is MIAME compliant and the raw data has been deposited in the Gene Expression Omnibus(GEO) database. For each gene we calculated either the ratio between the RPE and the photoreceptor signal, or the ratio between the RPE and the choroid signal, depending on the array. This resulted in three ratio's for the RPE versus photoreceptors and three ratio's for the RPE versus choroid for each gene. Only when all three ratio's for a single gene were greater than 2.5, we considered a gene to have a meaningfully higher gene expression (GE) in one tissue compared to the other. We chose differences in GE of at least two-fold (fold change (FC)>2.5) as cut off criterion for RPE compared to photoreceptor GE (RPE>phot), RPE compared to choroid GE (RPE>chor) and for RPE compared to photoreceptor as well as choroid GE (RPE>phot&chor). The same criteria were applied to photoreceptor compared to RPE GE (phot>RPE) and choroid compared to RPE GE (chor>RPE). A functional analysis of Kegg pathways (Kyoto Encyclopedia of Genes and Genomes) and functional categories was performed on all groups of genes with an average FC>2.5 using the David online software [11]. Cut off criteria used were a p-value of less than 0.001 using either a Benjamini-Hochberg correction or an Ease score, a modified Fisher's exact test [11], [24]. More advanced analyses of our RPE-specific genes was performed with the Ingenuity knowledge database [25] (version IPA 7.4, april 2009) yielding biological functions, canonical pathways and gene networks. We searched the literature for proof of RPE expression of our RPE-specific genes with high expression levels using the Genecards website [26] the online Mendelian inheritance in man (OMIM) website [27] and the Pubmed website [28] using the gene name combined with ‘RPE’ or ‘retina’ or ‘retinal pigment epithelium’ or ‘expression’ as search criteria.

### Confirmation of Microarray Results

For confirmation of our microarray data sQRT-PCR was used (and not QPCR) since sQRT-PCR is less sensitive for a) relatively poor RNA quality which is unavoidably obtained from human donor eyes, and b) for adjacent cell contamination of the LDM samples. Moreover, our aim was to find an approximation of expression in the RPE, choroid and photoreceptors.

sQRT-PCR was carried out, in triplicate, using exon spanning primers on RNA from LDM derived cell samples of the RPE, choroid and photoreceptors. Primer sequences are available on request. Six genes for which no further literature data on RPE gene expression was available, were tested (Table 4). B-actine, a household gene, was used to normalize gene expression in between all cells of the retina.

## Supporting Information

Text S1 All features on the array. All 33,712 features present on the array.
(2.32 MB XLS)

Text S2 All genes with higher expression in the RPE than the photoreceptors. List of all genes with expression levels 2.5 fold higher in RPE than in photoreceptors (average).
(0.18 MB XLS)

Text S3 All genes with higher expression in the RPE than the choroid. List of all genes with expression levels 2.5 fold higher in RPE than in choroid (average).
(0.11 MB XLS)

Text S4 All genes with RPE specific expression. List of all genes with expression levels 2.5 fold higher in RPE than in both choroid and the photoreceptors (average).
(0.05 MB XLS)
